# Enhancing Nursing Students’ International Competencies Through a Virtual Exchange Program: A Quasi-Experimental Study

**DOI:** 10.3390/nursrep16080258

**Published:** 2026-07-27

**Authors:** Tomomi Suda, Piyaorn Wajanatinapart, Ni Luh Putu Dewi Puspawati, Lisa Kerckhof, Sachiko Makabe, Anak Agung Istri Dalem Hana Yundari, Suparpit Maneesakorn von Bormann

**Affiliations:** 1 Department of Nursing, Akita University Graduate School of Health Sciences, Akita 010-8543, Japan; stomomi@hs.akita-u.ac.jp (T.S.); smaka@hs.akita-u.ac.jp (S.M.); 2 Institute of Nursing, Suranaree University of Technology, Nakhon Ratchasima 30000, Thailand; piyaorn@g.sut.ac.th; 3 Department of Nursing, Wira Medika Bali Health College, Denpasar 80239, Indonesia; dewipuspawati@stikeswiramedika.ac.id (N.L.P.D.P.); hanayundari@gmail.com (A.A.I.D.H.Y.); 4 Department of Healthcare, VIVES University of Applied Sciences, 8500 Kortrijk, Belgium; lisa.kerckhof@vives.be

**Keywords:** virtual, nursing, education, exchange program, intercultural competence

## Abstract

**Background/Objectives:** We conducted a virtual exchange program (VEP) to facilitate discussions on the Sustainable Development Goals (SDGs) among nursing students in Belgium, Indonesia, Japan, and Thailand and to assess the improvements in international competencies. **Methods:** In this quasi-experimental study with a pre- and post-test design, students who voluntarily participated in the VEP were compared with a matched group of non-participants. International competencies were assessed using the STRENCO (Strengthening multi-professional competencies in mental health) Competency Assessment Tool, a self-assessment tool. Hence, this analysis is based on self-reported data, which were analyzed using a linear mixed-effects model. **Results:** The analysis included 29 and 34 students in the intervention and control groups, respectively. A significant interaction effect between group and time was observed for intercultural competence (*p* = 0.004, 95% confidence interval (CI): [0.18, 0.87]), personal growth (*p* = 0.013, 95%CI: [0.10, 0.79]), and digital competence (*p* = 0.020, 95%CI: [0.09, 1.05]). In contrast, the interaction was not significant for language skills (*p* = 0.168, 95%CI: [−0.10, 0.58]), global engagement (*p* = 0.716, 95%CI: [−0.28, 0.40]), and international discipline learning (*p* = 0.058, 95%CI: [−0.02, 0.87]). **Conclusions:** This SDG-focused VEP involving nursing students from four countries across Europe and Asia provided a structured approach to international exchange education and may have enhanced intercultural competence, personal growth, and digital competence as components of international competencies. This study is expected to explore future VEP-based practices and international exchanges.

## 1. Introduction

In a rapidly diversifying modern society, developing international competencies, which enhance cultural awareness and promote respectful interactions with others, is crucial [1]. These competencies form the foundation of global competence by enabling individuals to effectively engage with diverse cultures and address complex global challenges. Global competence is defined as “a multidimensional construct that requires a combination of knowledge, skills, attitudes, and values to be successfully applied to global issues or intercultural situations” [2]. Grounded in this conceptual framework, such comprehensive competence can be operationalized through specific international competency domains. These include language skills, intercultural competence, global engagement, personal growth, international disciplinary learning, and digital competence [3,4]. These competencies allow individuals to explore local, global, and intercultural issues, understand and appreciate different perspectives and worldviews, and take responsible action to promote sustainability and collective well-being [2].

These competencies are essential in nursing; a profession grounded in both science and humanity. Nurses are required to understand the diverse cultural backgrounds of individuals and provide flexible and effective care [5]. Consequently, with the advancement of globalization, acquiring international competencies has become crucial in nursing. Furthermore, Shulman [6] suggests that professional education plays a key role in shaping students’ ways of thinking, acting, and valuing, and thus influences the development of competencies such as international competencies.

However, developing nursing students’ international competencies poses several challenges. Language skills, among the international competencies, are essential for effective communication. Nurses often face language barriers, which can lead to increased stress and workload when caring for patients who speak different languages, despite their desire to provide high-quality care regardless of language [7].

In addition to language issues, intercultural understanding is challenging. Simply understanding different cultures when practicing nursing in a multicultural environment has been reported as insufficient; rather, international experience, which enhances nursing practice skills needed to respond to cultural differences in behavior and attitudes, is crucial [8]. In the nursing curriculum, early exposure to and learning from international experiences provide a foundation for nursing students to enhance their international competencies and demonstrate them in practice [9].

International study programs provide a useful approach for developing nursing students’ international competencies. These short-term programs are preferred by nursing students, who often face a curriculum overloaded with structured academic requirements for licensure in their own countries [10]. However, because research indicates that the intercultural competence outcomes as part of international competencies of short-term study abroad programs do not match those of long-term programs, short-term programs require a more strategic and iterative approach to presenting intercultural content [11].

One way to address the limitations inherent to short-term study abroad interventions is the implementation of a virtual exchange program (VEP). A VEP is an alternative form of student exchange that offers the same benefits as traditional student mobility but utilizes more familiar tools and a lower-cost, as well as safer method of student exchange [12]. Having the advantage of reducing physical and financial burdens and enabling more students to participate, a VEP can be implemented repeatedly, even in an already crowded nursing education curriculum.

Although traditional study abroad has been the focus of numerous studies, research on virtual exchange is not yet comprehensive [13]. In 2020, a mixed-methods study evaluated global leadership competencies among 15 South Korean nursing students participating in a virtual international collaborative learning program with the United States [14]. The results indicated enhanced global leadership competencies in a single-group post-test design without a control group. In 2020, qualitative studies conducted in Hong Kong, Australia, and Sweden showed that online activities influenced the development of cultural awareness [15].

These results demonstrate that nursing students can accumulate international experience through VEPs. These programs can also be conducted repeatedly without the constraints of travel-related physical limitations. Previous research has predominantly been quantitative, with homogeneous group comparisons conducted only after the program [14,15]. By contrast, qualitative research often depends on participants’ personal experiences. Additionally, although research has found that specific international competencies, such as global leadership and intercultural understanding, improve through these programs [14,16], there is limited clarity regarding the comprehensive development of these diverse international competencies.

In this study, we aimed to identify improvements in the international competencies of nursing students from four countries through participation in a VEP. Specifically, this study exploratorily examines whether participating in the VEP would improve their international competencies across six domains as assessed by the STRENCO (Strengthening multi-professional competencies in mental health) [4] Competency Assessment Tool, compared to those who did not. The STRENCO Competency Assessment Tool was developed to evaluate the competencies required for international collaborative work and is intended for use as a reflective self-assessment. Although originally designed for the mental health field, its domains and items are not limited to targeted groups and include digital competencies essential for VEP, consistent with the aims of this study. This study is original in its comparison of six international competency domains in SDG-focused nursing education across four countries with a control group. This study is expected to explore future nursing educational prospects and examine VEP-based practices and international exchanges.

## 2. Methods

### 2.1. Study Design

We conducted a quasi-experimental study with a pre- and post-test design because randomization was not feasible due to the nature of the intervention. The intervention group included students who voluntarily participated in the VEP. The control group consisted of non-participating students matched to the intervention group based on academic year, sex, and country.

### 2.2. Virtual Exchange Program

**Figure 1:**
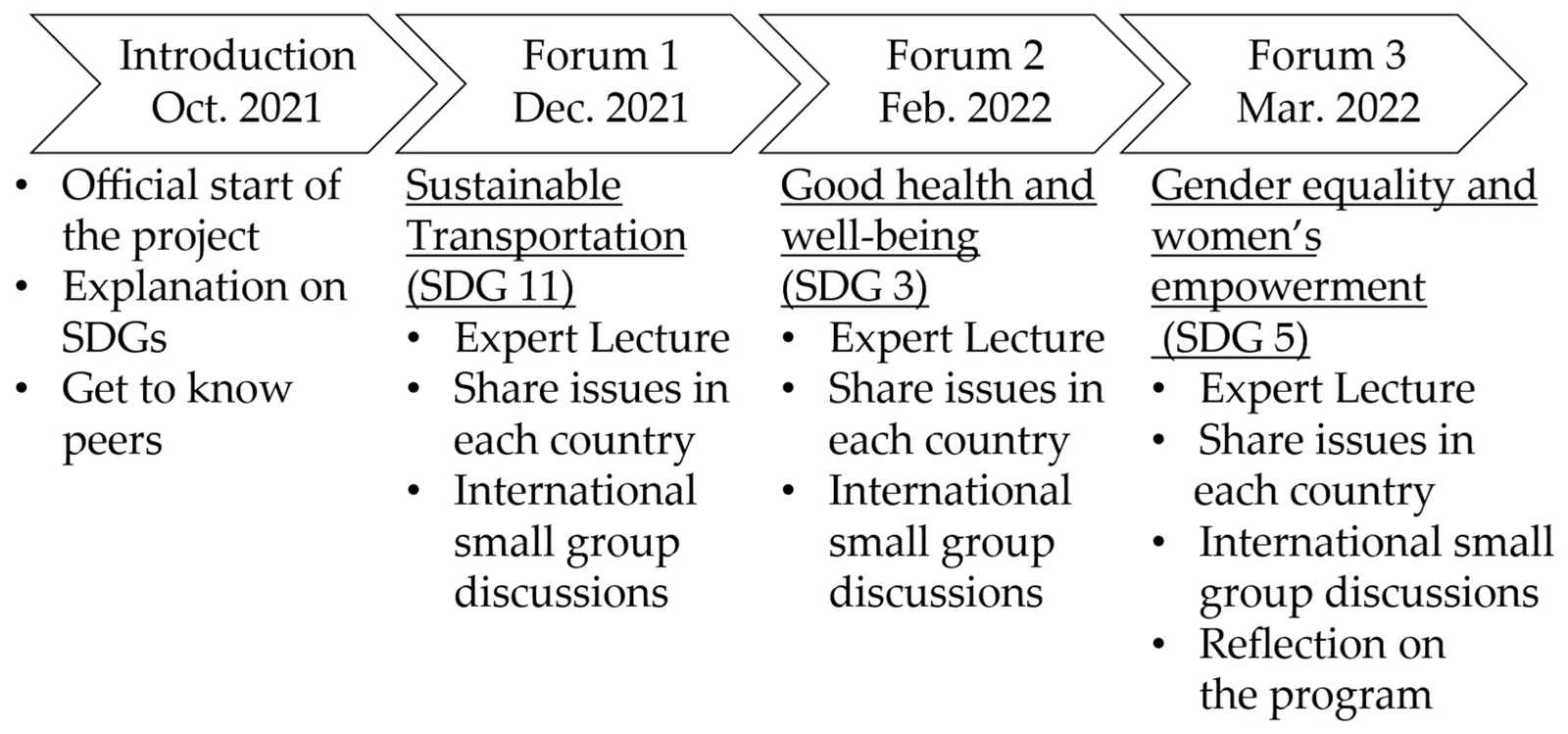
Virtual exchange program schedule.

We conducted an international exchange program in English involving Belgium (VIVES University of Applied Sciences, Kortrijk, Belgium), Indonesia (WIRA Medika Bali Health College, Bali, Indonesia), Japan (Akita University, Akita, Japan), and Thailand (Suranaree University of Technology, Nakhon Ratchasima, Thailand) using an online platform. These four countries have incorporated international nursing education into their curricula to educate nursing students to adapt to diverse cultural backgrounds. Nursing students participated in the program remotely. The project focused on the SDGs, dealing with community problems, and involved collaboration with international peers toward a common goal. The program was conducted over six months, from October 2021 to March 2022, for four sessions of two hours per session (Figure 1).

The program is based on Tuijnman and Boström’s [17] concept of lifelong education and adopts the pedagogical framework of virtual exchange demonstrated by Todhunter et al. [18]. This framework encompasses formal academic, formal social, informal academic, and informal social approaches, fostering knowledge development through interaction across these four quadrants, involving both light social interaction and formal academic structures [18]. The program is grounded in the Constructivist Theory of Learning [19], which posits that learning is an active, constructive process. Students actively construct knowledge through reflection, discussion, and collaboration with peers. This program was designed to include four quadrants.

In practice, various digital platforms were used: the sessions were held via Zoom, collaborative tasks were performed using Google Sheets, and out-of-session communication was conducted via WhatsApp or LINE, as it is widely used in East and Southeast Asia. Each session was facilitated alternately by two student representatives from each country, following preparatory student meetings and rehearsals with faculty members. In each session of VEP, participants received academic lectures from experts to share foundational knowledge, and subsequently, they presented their own country’s situation to international peers. Following these presentations, participants were divided into six small groups of 9–10 students each using the Zoom breakout room feature, ensuring an equal distribution of students from the four participating countries. The group composition was changed for each session, and students discussed ideas with their international peers in small groups to deepen their problem-solving and discussion abilities. English was used as the common language within these small groups; however, 2–3 faculty members from each country were allocated to each group to provide language support for non-native English speakers. Finally, each session ended with small-group presentations and reflections. Overall, an attendance rate of 90% was maintained throughout the program.

In addition to remote synchronous education within the program, students also engaged in remote asynchronous work to prepare for the program domestically and internationally. Specifically, students collaborated with their domestic peers outside of the sessions to prepare a 5–10 min presentation introducing their country’s context. Some participants also developed international friendships and maintained communication through collaborative work outside of the session. When nurses focus only on SDG 3, regarding good health and well-being, they risk having a narrow perspective in addressing complex and interrelated health and social needs [20]. We focused on various SDGs to examine the challenges influenced by the distinct social and cultural contexts of different countries, which can contribute to health. The second session focused on reinforcing efforts toward preventing road traffic deaths and injuries through sustainable transport (SDG 11). The third session focused on strengthening the prevention and treatment of substance abuse, including narcotic drug abuse and harmful use of alcohol, for the most vulnerable women and children in good health and well-being (SDG 3). The fourth session focused on decent work, eliminating gender wage gaps, gender equality, and women’s empowerment (SDG 5).

Based on the theoretical frameworks and intended learning objectives described above, the VEP was designed, and the relationship between program activities and assessed international competencies is shown in Table 1.

### 2.3. Participants

The total number of nursing students from the four countries was 674. The intervention group consisted of 55 students who participated in the VEP, including 11 from Belgium, 13 from Indonesia, seven from Japan, and 24 from Thailand. Among them, 29 students were assigned to the intervention group after voluntarily applying in response to recruitment posters sent via email. The 29 students responded to the pre-test and participated in VEP. After the VEP, a post-test was conducted, with two students dropping out due to non-response. Consequently, 27 participants completed both the pre- and post-tests.

The control group included 619 nursing students who did not participate in the VEP, namely 141 from Belgium, 241 from Indonesia, 197 from Japan, and 40 from Thailand. Following the allocation of the intervention group, among the eligible non-participants, control group participants were selected to match the intervention group based on the academic year, sex, and country. Participants were recruited through a two-stage process conducted via email. First, a recruitment invitation email was sent to all eligible non-participating students. Second, a follow-up email was sent to specific students to match the characteristics of the intervention group. Once this predefined target was achieved, recruitment was closed. Notably, 34 students responded to the pre-test. The post-tests for the control and intervention groups were administered concurrently; however, eight students dropped out due to non-response. Consequently, only 26 participants completed both the pre- and post-tests.

During the study period, international travel for the intervention and control groups was restricted due to COVID-19. The control group did not participate in the VEP; however, they received the same university education as the intervention group, including English and international nursing lectures, and the use of a distance learning system. Interaction between the two groups within university life was unrestricted; however, no activity reports regarding the VEP were shared with the control group.

**Figure 2:**
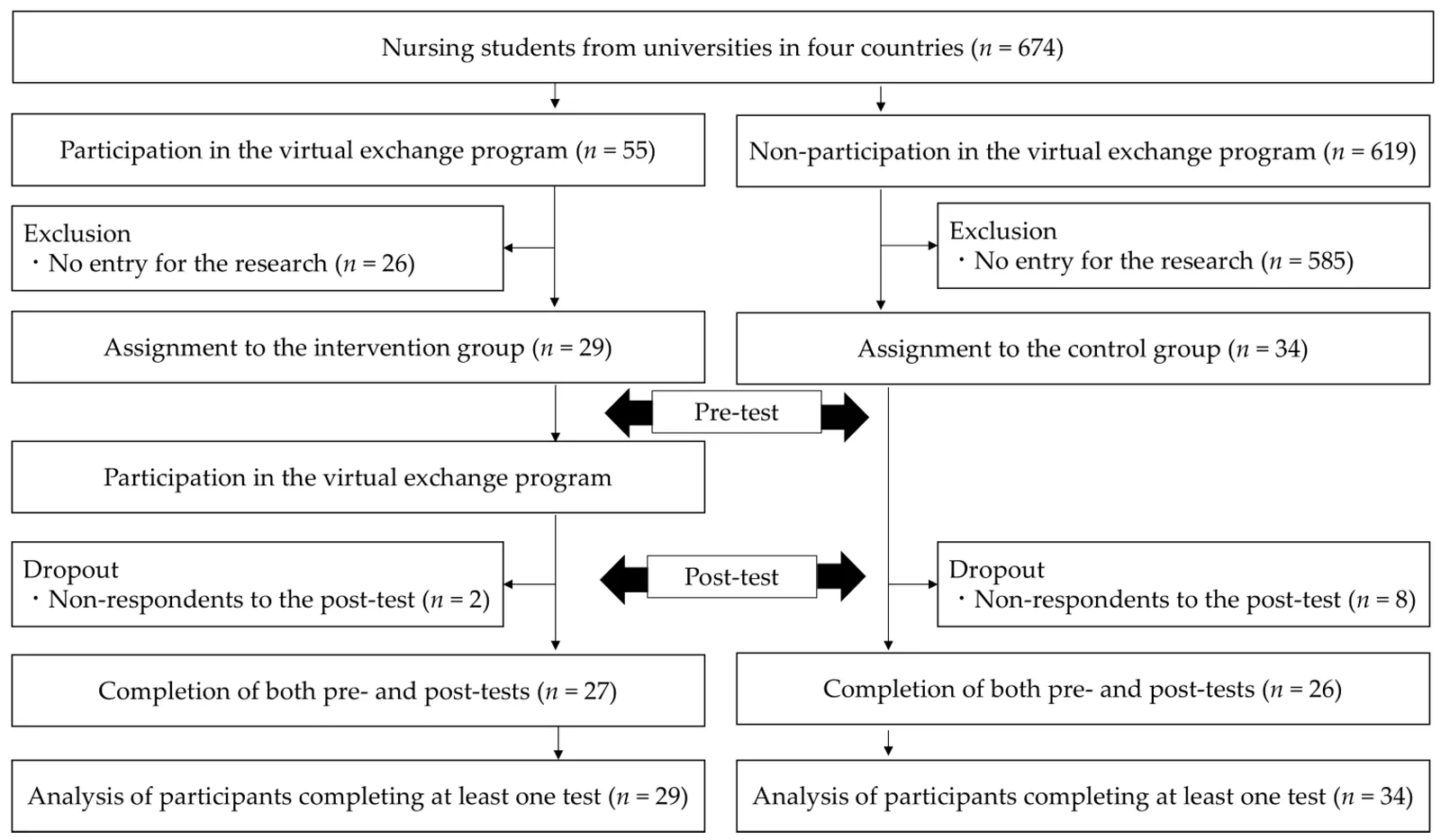
Flow diagram of the study participants.

G*Power 3.1.9.7 was used for sample size power calculation. For a repeated measurement analysis of variance for two groups with a medium effect size of 0.25, 0.05 α error, and 0.90 power, a sample size of 46 was needed. Based on the assumption of dropouts, the recruitment sample size was 55. However, in the post-test, two and eight participants were missing in the intervention and control groups, respectively. Data were analyzed using a linear mixed-effects model (LMM) to minimize the loss of information due to these missing values, with participants included as random effects to account for repeated measurements within individuals. The final number of participants analyzed was 29 and 34 in the intervention and control groups, respectively (Figure 2).

### 2.4. Data Collection

Data were collected twice using an English-language digital questionnaire via email for the pre-test during October–December 2021 and for the post-test in March 2022 for both the intervention and control groups. The questionnaire included demographic data and the STRENCO Competency Assessment Tool [4], which was used to measure international competencies. This scale includes six domains and 35 items rated on a 5-score Likert scale ranging from 1 (totally absent) to 5 (excellent). A high mean score for each domain indicates a perceived high competence in that domain. Domain 1 is “language skills” and includes four items: writing a text in another language, speaking a foreign language, being able to understand oral texts in a foreign language, and being able to understand a written text in a foreign language. Domain 2 is “intercultural competence” and includes nine items: cultural self-knowledge, flexibility, resilience, responsiveness, knowledge, connectivity competence, communicative competence, conflict management, and multi-perspective approach. Domain 3 is “global engagement” and includes four items: competence in international orientation, forming an own opinion regarding societal or international topics, expressing an own opinion on societal or international topics, and showing social involvement. Domain 4 is “personal growth” and includes eight items: being able to function independently, cooperating and networking, showing confidence, showing a flexible attitude, investigating other perspectives, showing creativity, possessing a clear idea of the future, and holding one’s own in stressful situations. Domain 5 is “international disciplinary learning” and includes four items: being able to situate their discipline within the international context, recognizing the fact that their domain is culturally determined, having developed knowledge of the professional activities of their discipline in other countries, and being aware of relevant international organizations within their field. Finally, Domain 6 is “digital competence” and includes six items: digital communication, creativity, innovation, citizenship, awareness, and working with digital content. Cronbach’s alpha of pre- and post-tests in this study was 0.97 in total. Cronbach’s alpha of each domain was calculated as follows: 0.86 for the pre-test and 0.75 for the post-test in Domain 1, 0.88 for the pre-test and 0.91 for the post-test in Domain 2, 0.83 for both the pre- and post-tests in Domain 3, 0.90 for both the pre- and post-tests in Domain 4, 0.77 for the pre-test and 0.83 for the post-test in Domain 5, and 0.87 for the pre-test and 0.93 for the post-test in Domain 6. The six international competency domains were pre-specified as the primary outcomes of this study.

### 2.5. Data Analysis

Demographic data were statistically analyzed. The normality of the data was assessed using the Shapiro–Wilk test. Descriptive statistics were presented as numbers, percentages, medians, and interquartile ranges. The Mann–Whitney U test and Fisher’s exact test were performed to assess the participants’ demographic characteristics in the intervention and control groups. The mean score and 95% confidence interval (CI) were calculated for each international competency. An LMM was used to compare pre- and post-test scores on the six international competencies between the intervention and control groups, with group, time, and their interaction included as fixed effects, and participant included as a random effect. This approach included a random intercept for each participant to account for individual differences, enabling the evaluation of changes in international competencies over time and differences between the groups. The LMM was adopted because it includes all participants who completed at least one test, and incorporates data from dropouts, thereby enhancing statistical power with a limited sample size. Due to limited sample sizes per country, cross-national analysis was not performed. Instead, data from all four nations were treated as a single sample to focus on the overall improvements in international competencies rather than on national variations. All statistical analyses were performed using R software, version 4.3.0 (R Core Team, 2023) [21], and the significance level was set at *p* < 0.05.

### 2.6. Ethical Considerations

This study was approved by the Research Ethics Committees of each institute: the Research Ethics Committees of VIVES University of Applied Sciences (2021-52), WIRA Medika Bali Health College (04.0557/KEPITEKES-BALI/XI/2021), Akita University (2749), and Suranaree University of Technology (EC-64-151). The participants voluntarily responded to the digital questionnaire after providing their consent to participate in the study.

## 3. Results

### 3.1. Demographic Characteristics of Participants

The study included 63 participants: 29 in the intervention group and 34 in the control group, all of whom were non-native English speakers. Most participants were female (*n* = 57, 90.5%), and most were first- or third-year students (*n* = 26, 41.3%). Most participants were from Indonesia (*n* = 20, 31.7%), followed by Belgium (*n* = 19, 30.2%), and Japan and Thailand (*n* = 12, 19.0%). Random assignment was not possible because of the open nature of the exchange program. The control group was recruited by matching academic year, sex, and country with the intervention group. To ensure baseline equivalence, the independent Mann–Whitney U test and Fisher’s exact test were performed, showing no significant differences between the two groups (Table 2).

Overall, 10 participants dropped out due to non-response to the post-test, including two in the intervention group (both third-year students from Belgium) and eight in the control group (two first-year students from Japan, five third-year students from Belgium, and one third-year student from Indonesia).

### 3.2. International Competencies

Table 3 shows the mean scores and 95%CIs of international competencies by group and attrition status. For all international competencies, the baseline mean scores of dropouts were higher than those of completers in both the intervention and control groups. Notably, some 95%CIs did not fully overlap; however, the 95%CIs for dropouts were wide and largely overlapped with those of the completers.

Table 4 shows the results of the LMM analysis for international competencies. A statistically significant interaction was observed between group and time for intercultural competence (Estimate = 0.53, *t* = 3.03, *p* = 0.004, 95%CI: [0.18, 0.87]), personal growth (Estimate = 0.44, *t* = 2.59, *p* = 0.013, 95%CI: [0.10, 0.79]), and digital competence (Estimate = 0.57, *t* = 2.40, *p* = 0.020, 95%CI: [0.09, 1.05]).

In contrast, the interaction between group and time was not statistically significant for language skills (Estimate = 0.24, *t* = 1.40, *p* = 0.168, 95%CI: [−0.10, 0.58]), global engagement (Estimate = 0.06, *t* = 0.37, *p* = 0.716, 95%CI: [−0.28, 0.40]), and international discipline learning (Estimate = 0.43, *t* = 2.59, *p* = 0.058, 95%CI: [−0.02, 0.87]).

**Figure 3:**
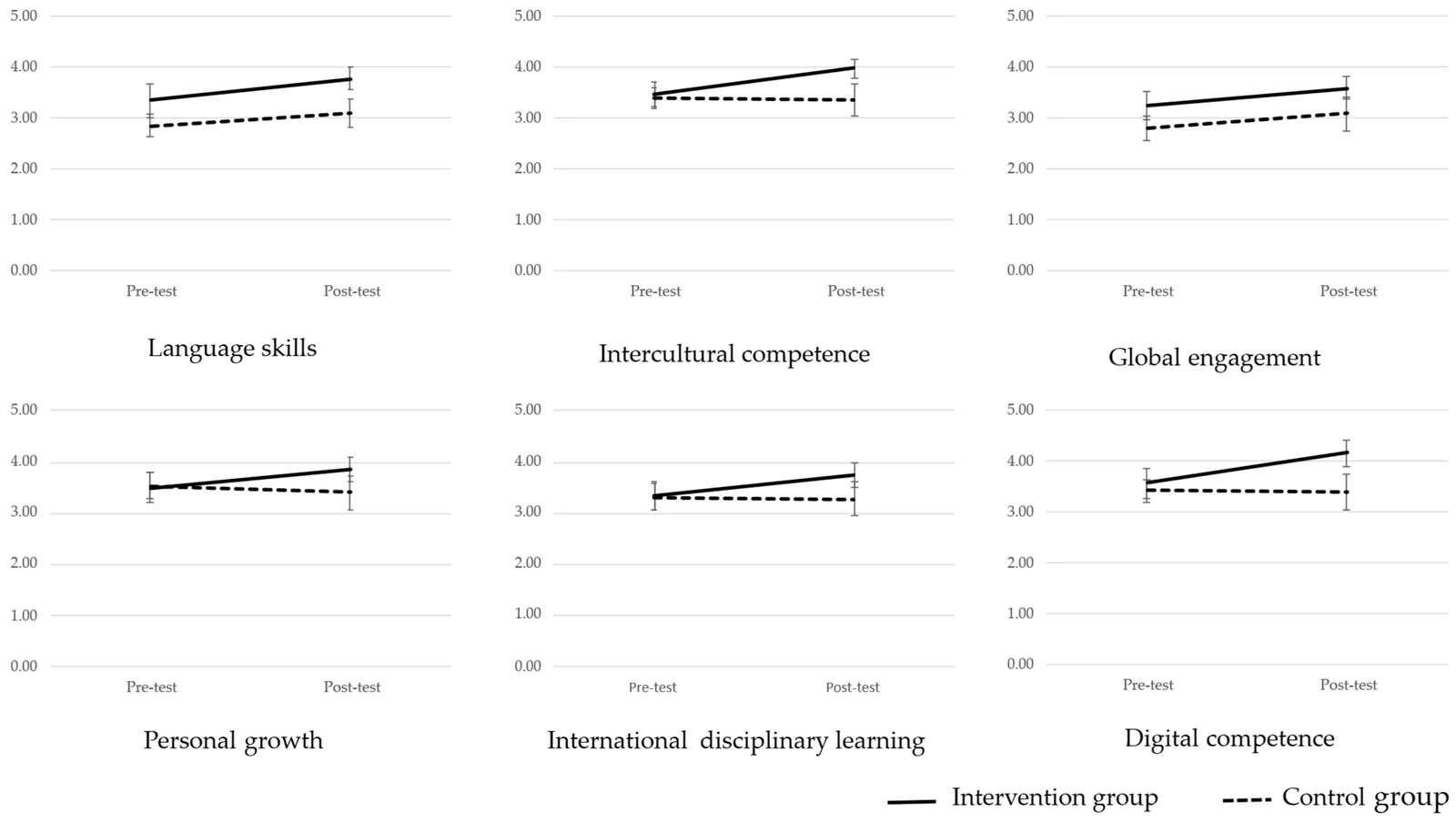
Mean scores with 95% confidence intervals for each international competency comparing the intervention and control groups at the pre- and post-test.

Figure 3 shows the mean scores with 95%CIs for each international competency, comparing the intervention and control groups at the pre- and post-test.

## 4. Discussion

This study suggests the effectiveness of a VEP in intercultural competence, personal growth, and digital competence of nursing students based on self-reported evaluations. No significant differences in other competencies, such as language skills, global engagement, or international disciplinary learning, were detected between the two groups. Although some of these results are consistent with previous studies [22,23] in suggesting that online exchange contributes to self-development, intercultural awareness, and collaborative engagement, some competencies showed different outcomes. This quasi-experimental study contributes previous research by suggesting the potential effectiveness of a VEP in nursing education across four countries spanning Europe and Asia. Nevertheless, the findings should be interpreted cautiously considering the limitations discussed below, including self-reported assessments, potential self-selection bias from voluntary VEP participation, and baseline differences between groups.

Regarding intercultural competence, personal growth, and digital competence, the intervention group showed improved post-test competence compared to the control group. Regarding intercultural competence, Hackett et al. [24] have conducted a quasi-experimental study to examine the effectiveness of collaborative online international learning among 108 undergraduate students in the United States and the Netherlands. The United States sample showed an increase in intercultural competence in the intervention group, particularly in terms of cultural intelligence. Previous qualitative studies on nursing students have also reported increased intercultural competence and cultural sensitivity among students who participated in such programs [16]. This study’s findings align with those of previous studies, suggesting that online education may improve intercultural competence. Nursing should not only address SDG 3, which focuses on health and well-being, but also understand complex issues beyond physical aspects and navigate diverse cultures with multiple legal frameworks [13,25]. Nursing needs to move beyond a traditional perspective focused on individuals and disease and adopt a broader perspective that encompasses social determinants such as education and economic stability [25]. In this study, VEP participants discussed various SDGs. The session reflections indicated that participants recognized that the differences in drug legality across countries are shaped by historical, cultural, and legal frameworks. Some participants also commented that they also realized that the regulations and beliefs they had been adhering to were not universal. Such reflective learning has been shown to enhance intercultural competence [26]. These findings suggest that intercultural competence may be enhanced through both recognition of one’s own culture and understanding of other cultures, which may contribute to nursing competencies required to care for individuals from diverse cultural backgrounds.

In a previous study of personal growth, the collaborative process of Serbian and American students in a virtual space highlighted the challenges of personal growth in intercultural exchanges, indicating the need to consider not only the achievement of collaborative goals but also the development of personal growth and a sense of community [27]. In the VEP of this study, the participants were provided with an environment in which they had to take the initiative, although in some situations, language barriers during small-group discussions and question-and-answer exchanges prevented them from working together. By challenging themselves and overcoming difficulties, participants may have improved their personal growth through repeated attempts to understand others and through self-reflection.

Regarding digital competence, the enhancement expected through VEP participation was observed, consistent with previous research [28]. During the pandemic, both groups had increased reliance on online platforms; however, in the VEP of this study, engaging in the international co-creation of digital content using collaborative tools, alongside the creative and innovative use of digital features in presentations, may have contributed to improvements, as digital competence is essential to the VEP nature.

No significant differences were observed in language skills, global engagement, or international disciplinary learning between the intervention and control groups. In particular, as the control group also showed improvements in language skills and global engagement during the post-test, these findings suggest that, compared to the VEP, alternative educational approaches may also contribute to the development of these competencies. Regarding language skills in this study, the intervention group spent more time using a non-native language during various activities, both inside and outside the sessions. However, in previous studies, it has been reported that, in second language processing, limited language knowledge and associated anxiety can inhibit effective communication [29], and that self-efficacy plays an important role [30]. Therefore, further support is required for students to maintain their self-efficacy within the VEP as they accumulate successful experiences in expressing their opinions among peers despite language barriers. Furthermore, as the control group also showed improvements in post-test scores, the findings suggest that language skills may also be enhanced through other language learning. Regarding global engagement, research has indicated that the intensity of study abroad programs have a long-term impact on this competency [31]. In addition to enhancing VEP content, students may further develop this competency by combining the VEP with experiences such as short-term study abroad programs, in which they directly engage with social and international issues in other countries. Furthermore, as the control group also showed improvements in post-test scores, improvements in global engagement may have been related to increased exposure to international topics during the COVID-19 pandemic within this study period. Regarding international professional learning, the SDGs were featured as the main theme of this VEP. Previous research indicated that nurses may find it challenging to incorporate the SDGs into their clinical roles [13,25]. Therefore, supporting students in recognizing transnational issues as personally relevant to nursing is also crucial for broadening their perspectives.

The findings encourage further consideration of implementing similar interventions in nursing education to improve international competencies, especially personal growth, intercultural competence, and digital competence. The VEP offers advantages, such as eliminating exchange barriers caused by time, distance, and financial constraints inherent to traditional exchanges, making it easier to interact with international health colleagues [32]. However, there are also difficulties in implementing the VEP. Indeed, although virtual exchange can provide a knowledge-based component, achieving transformative experiences and sufficient cultural development comparable to traditional study abroad programs remain challenging [33], indicating the importance of carefully structured program content.

Furthermore, these international competencies are developed through a continuous process rather than as the direct result of a single experience [34]. Previous studies have suggested that combining the VEP with English audiovisual activities, reading, and live communication, and incorporating online pre-departure preparation before studying abroad, can stimulate intercultural learning [35]. Future studies should explore ways in which the VEP can be combined with traditional study abroad programs to complement its limitations and provide students with a wide range of international exchange opportunities. In addition to traditional study abroad and language learning experiences, the VEP should be integrated into educational approaches to enrich the overall learning experience and support the continued development of international competencies. Therefore, the findings might be used to revise the program, integrate it with traditional study abroad approaches, and explore potential mechanisms underlying improvements in international competencies.

### Study Limitations

This study has some limitations. First, its design was quasi-experimental study and it was conducted in accordance with the program’s characteristics. There may be differences in international competency improvement and baseline scores between the intervention group, which voluntarily participated in the VEP, and the control group, which did not participate; therefore, self-selection bias cannot be ruled out. Wong et al. [36] found that individuals with higher levels of cultural self-efficacy, intercultural communication competence, perceived social competence, individual interest, foreign language learning motivation, and perceived value of the program had stronger intention to participate. In this study, the participants in the intervention group had higher pre-test scores in some competencies than those in the control group, which may have influenced the results. Furthermore, 16% of the small sample dropped out before the post-test, and the number of these dropouts varied between the groups; however, these dropouts were still included in the LMM analysis. Larger sample sizes are needed. In addition, various contextual factors such as baseline international competency levels and different cultural backgrounds should be considered. Nevertheless, we compared the intervention and control groups with respect to reliance on online platforms during a period when international travel—one of the factors influencing international competencies—was restricted due to the pandemic.

Other limitations of this study include unclear processes for developing international competencies and the inability to identify the long-term impact of the VEP because assessments were only completed pre- and post-program. Therefore, further qualitative, mixed-methods, and longitudinal research focusing on the experiences of diverse participants is needed to explore how experiences during VEP contribute to the developmental processes of international competencies and how these are applied in continuous learning and practice.

In addition, the STRENCO competency assessment tool was treated as a measurement scale and analyzed by domain. This scale was validated and examined by mental health experts from five countries using the Delphi method; however, its reliability and validity have not yet been published in academic journals. In this study, it was adopted based on its suitability for the research objectives and the results of a preliminary trial conducted the previous year. Additionally, since the English version of the questionnaire was used, linguistic factors may have influenced the responses of participants whose native language is not English. As it is designed for self-assessment, the analysis is based on self-reported data and has inherent limitations. During the study period, the intervention group did not share VEP activity reports with the control group, which created the possibility of information contamination within the same university. However, the findings still provide valuable insights into the development of international competencies in nursing, although the use of objective measures, such as language tests or peer evaluations, may yield different results. The generalizability of the findings is limited due to the lack of detailed data on intervention implementation and adherence, and by the absence of long-term follow-up and verification of country-level variations. To address these limitations, further analyses are needed using multiple assessment methods that measure the long-term development of students’ international competencies.

## 5. Conclusions

The VEP for nursing students from four countries, including those from Europe and Asia, with discussions on the SDGs, offers a structured approach to international exchange education. This program was suggested to have enhanced intercultural competence, personal growth, and digital competence as components of international competencies based on self-reported evaluations. Furthermore, the VEP might provide valuable opportunities for intercultural learning and collaboration and address physical and economic barriers that could prevent nursing students from participating in traditional mobility programs. Future research should investigate the mechanisms by which international competencies develop.

## Figures and Tables

**Table 1:** Relationship between virtual exchange program activities and assessed international competencies.

International Competency	Learning Focus/Gap	Virtual Exchange Program Activity	Expected Learning Outcome	Measurable Outcome
Language skills	Limited experience communicating in a non-native language	Participation in non-native language discussions and activities	Communication in a non-native language through repeated opportunities to express opinions	Presentation text and delivery, discussion frequency and content, and understanding of lectures and debates
Intercultural competence	Limited opportunities to understand cultural differences and diversity	Analyzed their own country’s situation and participated in small-group SDG discussions with international peers	Recognition of one’s own cultural assumptions and increased understanding of other cultures	Analysis based on cultural knowledge, discussion based on a multi-perspective approach, adaptation of communication style, and acceptance of others
Global engagement	Limited engagement with international and societal issues	Attended expert lectures and discussed SDG-related global and community health issues	Formation and expression of own opinions on international and societal issues	Formation of own opinions through analysis within an international context, and expression of views incorporating proposals for societal solutions
Personal growth	Limited opportunities to develop independence, collaboration, and self-reflection in intercultural contexts	Small-group discussions with international peers fostering initiative and collaboration across language and cultural barriers	Development of independence, collaboration, and self-reflection through repeated attempts to understand others	Verbal frequency, active engagement, independence of task completion, team collaboration, and Self-reflection content
International disciplinary learning	Limited understanding of nursing roles in international contexts	Discussions on SDGs beyond good health and well-being (SDG 3), along with understanding healthcare systems and legal frameworks in each country to consider the role of nursing	Recognition of nursing roles within global, cross-national, and legally diverse contexts	Analysis of international nursing practices, understanding of cultural influences on nursing, and comparison of legally diverse nursing contexts
Digital competence	Limited experience in digital communication and collaboration in international settings	Participation in online synchronous and asynchronous activities	Use of digital tools for international communication and collaboration	Co-creation of digital content using collaborative tools, and creative and innovative use of digital features in presentations

Note: SDG = Sustainable Development Goals.

**Table 2:** Demographic characteristics of the participants.

	Total	Intervention Group	Control Group	*p*-Value
	(*n* = 63)	(*n* = 29)	(*n* = 34)	
Age	20	19	20	0.386 ^a^
Median (IQR)	(19.0–21.0)	(19.0–21.0)	(19.0–21.0)	
Gender				
Female	57 (90.5%)	26 (89.7%)	31 (91.2%)	1 ^b^
Male	6 (9.5%)	3 (10.3%)	3 (8.8%)	
Year of study				
1	26 (41.3%)	13 (44.8%)	13 (38.2%)	0.801 ^b^
2	4 (6.3%)	2 (6.9%)	2 (5.9%)	
3	26 (41.3%)	10 (34.5%)	16 (47.1%)	
4	7 (11.1%)	4 (13.8%)	3 (8.8%)	
Country of origin of the university				
Belgium	19 (30.2%)	8 (27.6%)	11 (32.4%)	0.941 ^b^
Indonesia	20 (31.7%)	10 (34.5%)	10 (29.4%)	
Japan	12 (19.0%)	5 (17.2%)	7 (20.6%)	
Thailand	12 (19.0%)	6 (20.7%)	6 (17.6%)	

IQR = Interquartile Range. Statistical significance was set at *p* < 0.05. ^a^ Determined by the Mann–Whitney U test. ^b^ Determined by Fisher’s exact test.

**Table 3:** Mean scores and 95% confidence intervals of international competencies by group and attrition status.

		Intervention Group, Mean (95%CI)			Control Group, Mean (95%CI)		
		Total	Completers	Dropouts	Total	Completers	Dropouts
Competencies		(*n* = 29)	(*n* = 27)	(*n* = 2)	(*n* = 34)	(*n* = 26)	(*n* = 8)
Language skills	Pre	3.34 (3.01–3.67)	3.25 (2.92–3.59)	4.50 (1.32–7.68)	2.85 (2.62–3.08)	2.85 (2.57–3.13)	2.88 (2.43–3.32)
	Post	3.77 (3.55–3.99)	-	-	3.10 (2.82–3.38)	-	-
Intercultural competence	Pre	3.45 (3.20–3.70)	3.41 (3.15–3.67)	4.06 (1.94–6.17)	3.39 (3.19–3.59)	3.31 (3.08–3.53)	3.67 (3.22–4.12)
	Post	3.96 (3.76–4.16)	-	-	3.36 (3.05–3.67)	-	-
Global engagement	Pre	3.25 (2.98–3.52)	3.19 (2.91–3.46)	4.13 (−0.64–8.89)	2.80 (2.55–3.06)	2.76 (2.46–3.05)	2.94 (2.31–3.57)
	Post	3.58 (3.36–3.80)	-	-	3.09 (2.76–3.43)	-	-
Personal growth	Pre	3.50 (3.21–3.79)	3.44 (3.13–3.74)	4.31 (0.34–8.28)	3.53 (3.27–3.79)	3.42 (3.11–3.73)	3.88 (3.41–4.34)
	Post	3.85 (3.62–4.08)	-	-	3.40 (3.06–3.74)	-	-
International disciplinary learning	Pre	3.34 (3.07–3.61)	3.32 (3.04–3.61)	3.50 (−2.85–9.85)	3.32 (3.05–3.59)	3.22 (2.90–3.54)	3.63 (3.03–4.22)
	Post	3.75 (3.51–4.00)	-	-	3.27 (2.93–3.61)	-	-
Digital competence	Pre	3.57 (3.27–3.87)	3.53 (3.22–3.85)	4.08 (0.91–7.26)	3.42 (3.21–3.63)	3.27 (3.04–3.50)	3.90 (3.45–4.34)
	Post	4.15 (3.91–4.40)	-	-	3.40 (3.05–3.75)	-	-

CI = confidence interval.

**Table 4:** Linear mixed-effects model results for international competencies.

Competencies	Source	Estimate	SE	*t*-Value	*p*-Value	95%CI	
						Lower	Upper
Language skills	Intercept	3.82	0.13	28.63	<0.001 ***	3.55	4.09
	Group	−0.72	0.19	−3.84	<0.001 ***	−1.10	−0.35
	Time	−0.48	0.12	−4.04	<0.001 ***	−0.72	−0.24
	Group × Time	0.24	0.17	1.40	0.168	−0.10	0.58
Intercultural competence	Intercept	3.98	0.12	33.35	<0.001 ***	3.75	4.22
	Group	−0.59	0.17	−3.47	0.001 **	−0.92	−0.25
	Time	−0.53	0.12	−4.32	<0.001 ***	−0.78	−0.28
	Group × Time	0.53	0.17	3.03	0.004 **	0.18	0.87
Global engagement	Intercept	3.62	0.14	26.61	<0.001 ***	3.35	3.90
	Group	−0.51	0.19	−2.67	0.010 *	−0.89	−0.13
	Time	−0.37	0.12	−3.10	0.003 **	−0.62	−0.13
	Group × Time	0.06	0.17	0.37	0.716	−0.28	0.40
Personal growth	Intercept	3.89	0.14	27.72	<0.001 ***	3.61	4.17
	Group	−0.41	0.20	−2.10	0.040 *	−0.81	−0.02
	Time	−0.39	0.12	−3.22	0.002 **	−0.63	−0.15
	Group × Time	0.44	0.17	2.59	0.013 *	0.10	0.79
International disciplinary learning	Intercept	3.75	0.14	26.41	<0.001 ***	3.47	4.04
	Group	−0.45	0.20	−2.23	0.030 *	−0.85	−0.05
	Time	−0.42	0.16	−2.67	0.010 *	−0.73	−0.10
	Group × Time	0.43	0.22	1.94	0.058	−0.02	0.87
Digital competence	Intercept	4.16	0.14	30.09	<0.001 ***	3.89	4.44
	Group	−0.73	0.20	−3.68	<0.001 ***	−1.12	−0.33
	Time	−0.59	0.17	−3.49	0.001 **	−0.94	−0.25
	Group × Time	0.57	0.24	2.40	0.020 *	0.09	1.05

* *p* < 0.05, ** *p* < 0.01, ***p < 0.001. SE = Standard error; CI = confidence interval.

## Data Availability

The data are not publicly available due to ethical restrictions. The participants were informed that the collected data would be used solely for research purposes. The data presented in this study are available on reasonable request from the corresponding author.

## Public Involvement Statement

No public involvement in any aspect of this research.

## Guidelines and Standards Statement

This manuscript was drafted in accordance with the TREND Statement (Transparent Reporting of Evaluations with Nonrandomized Designs) for a quasi-experimental study.

## Abbreviations

The following abbreviations are used in this manuscript:

VEP	Virtual exchange program
SDGs	Sustainable development goals
STRENCO	Strengthening multi-professional competencies in mental health
LMM	Linear mixed-effects model
CI	Confidence interval
